# Optic disc melanocytoma: a case report

**DOI:** 10.22336/rjo.2021.18

**Published:** 2021

**Authors:** Mannat Giran, Meenakshi Sindhu, Navya Naveen Kalra, Subina Narang

**Affiliations:** * Department of Ophthalmology, Government Medical College and Hospital, Chandigarh, India

**Keywords:** melanocytoma, benign ocular lesion, choroidal melanoma

## Abstract

Often misdiagnosed as melanoma, melanocytoma of the optic disc is a rare benign ocular lesion that requires minimal active intervention, but demands a life time follow-up.

We present a case of a 32-year-old man who was referred to our institute for the management of choroidal melanoma of the optic disc, which was detected by chance when the patient presented to a general ophthalmologist with chief complaint of itching in both eyes. The patient had normal visual acuity and fundoscopy revealed classical optic disc melanocytoma. The ancillary tests confirmed the diagnosis. The patient was kept under follow-up for four years, which showed no increase in size of the lesion.

The purpose of this presentation was to highlight the identifying features of ocular melanocytoma and differentiate it from other conditions requiring urgent intervention.

## Introduction

The term “melanocytoma” was first used in 1962 by Zimmermann to describe a benign, asymptomatic hamartomatous tumor that arises from melanocytes [**1**]. It needs to be differentiated from the other lesions of the optic disc in view of the grossly varied treatment and prognosis of these conditions. It is prudent to identify and differentiate benign melanocytoma from the malignant melanoma. 

We present a brief review of the features of melanocytoma to prevent unnecessary surgical intervention in these benign lesions.

## Case report

A 32-year-old Indian male was referred to the ocular oncology services of our institute for brachytherapy of eye tumor. The patient presented with chief complaints of mild itching in both eyes, when there was a chance detection of right optic disc choroidal melanoma by the ophthalmologist and he was referred to a tertiary care institute for further management. 

On ocular examination at our institution, his visual acuity was found to be 20/ 20 with normal pupillary reactions in both eyes. The intraocular pressures were 12 and 14 mmHg in right and left eye respectively. The slit lamp biomicroscopic examination revealed unremarkable anterior segments of both eyes, except for mild papillary hypertrophy in both palpebral conjunctivae. Right eye fundoscopy showed clear media with a black to dark brown elevated mass lesion, approximately one disc diameter, with feathery margins and was obscuring the optic disc. There was no evidence of any associated orange pigment, subretinal fluid, retinal edema, or disc edema. The left eye was essentially normal.

**Fig. 1:**
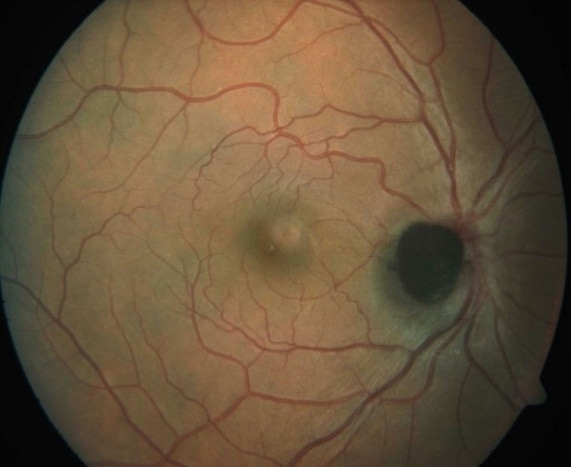
Fundus picture showing well-defined black to brown elevated lesion with feathery margins about one disc diameter in size obscuring the optic disc

There was no serous retinal detachment. B scan ultrasonography of the right eye confirmed a hyperechoic small dome shaped lesion, 1.5 mm in thickness with 1.8 x 1.8 mm base over the disc. Fundus autofluorescence showed the lesion to be hypoautofluorescent. Fundus fluorescein angiography revealed hypo-fluorescence in all stages without any evidence of late phase hyper-fluorescence of the mass lesion. There was staining of the disc margins nasally. The visual fields 24’2 showed enlargement of blind spot.

**Fig. 2:**
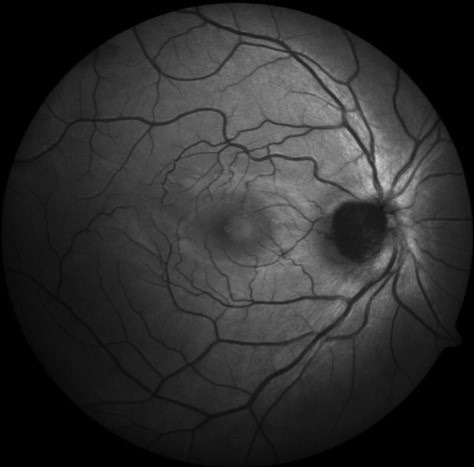
The legend is hypoautofluorescent on fundus autofluorescence

**Fig. 3 a,b:**
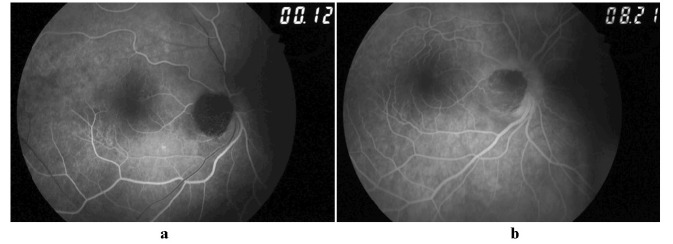
Fundus fluorescent angiography: the early and late frames show the lesion to be hypofluorescent

The clinical diagnosis of optic disc melanocytoma was made due to its characteristic location and clinical features and the patient was kept on a monthly follow-up for three months and six months there-after for four years. The patient maintained visual acuity of 20/ 20 without any increase in size of the lesion or alterations in visual fields. 

Patient was kept on regular follow-up with the help of ancillary tests like fundus photography and visual fields. At four years follow-up, the patient maintained the vision of 20/ 20 without any significant increase in size of melanocytoma or any alteration in the visual fields. 

## Discussion

Optic disc melanocytoma is typically less than 2 mm raised darkly pigmented mass lesion, which is known to be stationary or show minimal growth [**2**]. Melanocytoma classically involves the optic disc and in 18% of the patients it remains confined to the optic nerve head, while 77% cases have retina involvement, and 47% have choroidal involvement [**2**,**3**].

It is generally associated with good visual acuity and the vision of ≥ 20/ 40 is preserved in 93% of the cases [**2**]. The growth of the tumor is found to be in 11% of the cases at 5 years and in 32% at 10 years from the diagnosis [**2**,**4**]. In a study on the Korean population, the tumor growth was 0% at 1 year, 14% at 5 years, and 57% at 8 years [**5**]. The risk factors for the increase in size include increased tumor thickness, raised nodular configuration and tumor vascularization. The malignant transformation is seen in only 1-2% of the cases [**4**]. 

Melanocytomas could rarely have poor visual acuity, which is associated with compression on the optic nerve, exudative retinal detachment, choroidal neovascularization, tumor necrosis, central retinal vein obstruction and malignant transformation of the tumor [**7**]. Relative afferent pupillary defect was present only in those with poor visual acuity. The common visual field defects include the enlargement of the blind spot and nerve fiber bundle defects. The blind spot enlargement is related to the amount of tumor extension beyond the disc margin [**2**].

Optic disc melanocytomas are relatively avascular lesions and exhibit hypofluorescence with both indocyanine green and fluorescence [**2**,**4**]. A small area of hyperfluorescence observed in our patient was indicative of mild edema of the optic disc [**4**].

Melanocytomas have endogenous melanin and lipofuscin that transiently emit light (autofluorescence) when exposed to a light source. However, in comparison with a malignant melanoma, they show hypoautofluorescence in FAF as seen in our patient [**2**,**4**,**8**]. Newer imaging modalities like Infrared autofluorescence (IRAF) and short wave autofluorescence (SWAF) have shown greater sensitivity in identifying melanocytomas [**8**]. 

On B-scan ultrasonography, melanocytoma appears in a dome shaped configuration with medium to high internal reflectivity [**2**,**9**]. Gologorsky et al. employed high resolution B-scan to assess the benign lesion for signs of malignant transformation, such as increase in size, vascularity, and conversion to nodular appearance [**9**]. CT and MRI may not help much as the melanoma and melanocytoma appear practically the same in this modality [**2**].

**Fig. 4:**
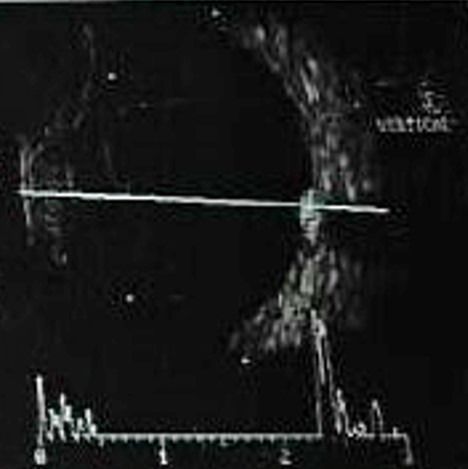
B-scan ultrasonography axial scan showing a dome shaped mass lesion over optic disc with high echogenicity

**Fig. 5:**
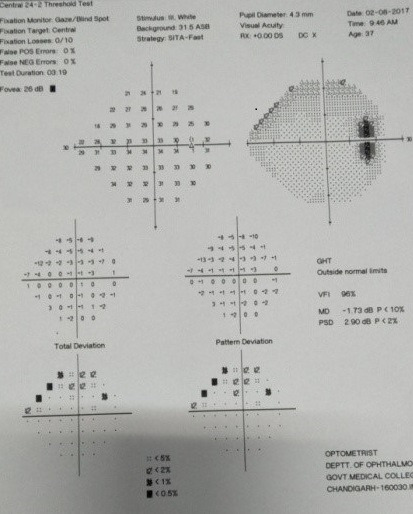
Computerized perimetry shows increased blind spot in the same eye

**Fig. 6:**
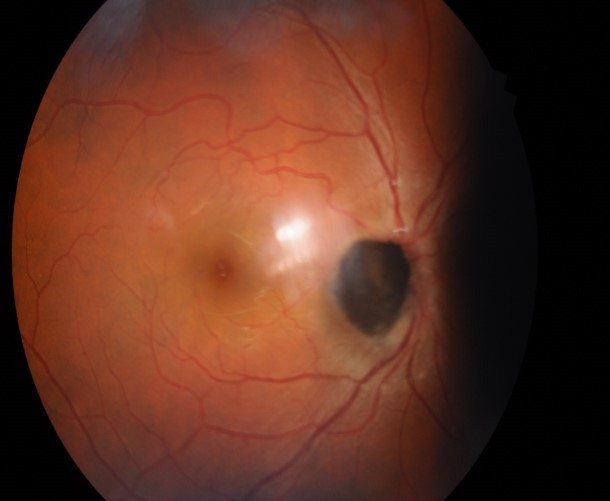
Fundus picture at 48 months follow-up with no increase in size and character of the mass lesion

It is important to differentiate melanocytoma from malignant melanoma. Malignant melanoma is characterized by a vascular mass lesion with orange pigmentation suggestive of lipofuscin, which is hyper-autofluorescent. It is usually more than 2 mm in thickness on echography and may show vascularization and serous detachment [**2**,**6**]. Other differentials of pigmented lesions of the disc are adenoma of retinal pigment epithelium, which is slightly off-center from the optic disc and does not show feathery margins. Optic disc invasion in malignant melanoma is seen in 5-7% of juxtapapillary tumors. The metastatic melanoma of the optic disc will show rapid growth and could infiltrate the optic disc and could give the appearance of papilloedema or papillitis. Choroidal naevus rarely involves the optic disc and naevi are usually flat with minimal elevation [**2**].

Optic disc melanocytoma requires a periodic lifelong follow-up as malignant transformation is known. At each follow-up visit it is essential to document and look for any change in size, shape, and consistency of the lesion.

## Conclusion

 The present case highlighted the importance of identifying optic disc melanocytoma. These cases do not require treatment and only require long-term follow-up to exclude malignant transformation.

**Conflict of Interest**

Authors state no conflict of interest.

**Informed Consent and Human and Animal Rights**

Informed consent has been obtained from all individuals included in this study.

**Authorization for the use of human subjects**

Ethical approval: The research related to human use complies with all the relevant national regulations, institutional policies, is in accordance with the tenets of the Helsinki Declaration, and has been approved by the Ethics Committee of Government Medical College and Hospital, Chandigarh, India.

**Acknowledgements**

None.

**Sources of Funding**

None.

**Disclosures**

None.
